# Insights into the pathophysiology of DFNA44 hearing loss associated with *CCDC50* frameshift variants

**DOI:** 10.1242/dmm.049757

**Published:** 2023-08-17

**Authors:** María Lachgar-Ruiz, Matías Morín, Elisa Martelletti, Neil J. Ingham, Lorenzo Preite, Morag A. Lewis, Luciana Santos Serrão de Castro, Karen P. Steel, Miguel Ángel Moreno-Pelayo

**Affiliations:** ^1^ Wolfson Centre for Age-Related Diseases, King's College London, Guy's Campus, London SE1 1UL, UK; ^2^ Servicio de Genética, Hospital Universitario Ramón y Cajal, IRYCIS and Biomedical Network Research Centre on Rare Diseases (CIBERER), km 9.100, 28034 Madrid, Spain

**Keywords:** Progressive hearing loss, Deafness, Next-generation sequencing, DFNA44, *CCDC50*, Ymer, Mouse mutant

## Abstract

Non-syndromic sensorineural hearing loss (SNHL) is the most common sensory disorder, and it presents a high genetic heterogeneity. As part of our clinical genetic studies, we ascertained a previously unreported mutation in *CCDC50* [c.828_858del, p.(Asp276Glufs*40)] segregating with hearing impairment in a Spanish family with SNHL associated with the autosomal dominant deafness locus DFNA44, which is predicted to disrupt protein function. To gain insight into the mechanism behind DFNA44 mutations, we analysed two *Ccdc50* presumed loss-of-function mouse mutants, which showed normal hearing thresholds up to 6 months of age, indicating that haploinsufficiency is unlikely to be the pathogenic mechanism. We then carried out *in vitro* studies on a set of artificial mutants and on the p.(Asp276Glufs*40) and p.(Phe292Hisfs*37) human mutations, and determined that only the mutants containing the six-amino-acid sequence CLENGL as part of their aberrant protein tail showed an abnormal distribution consisting of perinuclear aggregates of the CCDC50 protein (also known as Ymer). Therefore, we conclude that the CLENGL sequence is necessary to form these aggregates. Taken together, the *in vivo* and *in vitro* results obtained in this study suggest that the two identified mutations in *CCDC50* exert their effect through a dominant-negative or gain-of-function mechanism rather than by haploinsufficiency.

## Teaser

**Summary:** Analyses of *CCDC50* mutations in mice and cultured cells suggest that the mechanism of pathogenesis underlying human DFNA44 deafness is likely a dominant-negative or gain-of-function effect.

## INTRODUCTION

Sensorineural hearing loss (SNHL) is the most prevalent sensory disorder, affecting one to two in 1000 newborns worldwide, and genetic factors account for over half of the cases (Morton and Nance, 2006). Hereditary hearing impairment is characterised by a high clinical and genetic heterogeneity, with 124 genes identified so far in non-syndromic hearing loss (https://hereditaryhearingloss.org/) and as many as 1000 genes predicted to be involved (Ingham et al., 2019). The contribution of each gene towards the prevalence of SNHL may vary between different populations, and mutational screening needs to be conducted to establish this prevalence.

Among the genes involved in non-syndromic autosomal dominant SNHL is *CCDC50* (MIM 611051) (Vazza et al., 2003; Modamio-Høybjør et al., 2007), which maps to chromosome 3q28-29 within the critical interval of the autosomal dominant deafness locus DFNA44 (Modamio-Høybjør et al., 2003). *CCDC50* encodes coiled-coil domain-containing protein 50, also known as Ymer (Blagoev et al., 2004), and it is organised on 12 exons. Two alternative splice variants have been described: the longer isoform (NM_178335.3; NP_848018.1) is 482 amino acids (aa) long, and the shorter transcript (NM_174908.4; NP_777568.1), comes from alternative splicing that skips exon 6 and encodes a 306-aa-long protein. *CCDC50* is highly conserved across primates and rodents (Vazza et al., 2003). The mouse ortholog gene *Ccdc50* also has two isoforms [transcript variant 1 (NM_026202.4; NP_080478.2) a 305-aa-long isoform, and transcript variant 2 (NM_001025615.3; NP_001020786.1), a 290-aa-long isoform] and both are expressed in the mouse cochlea at different developmental stages [embryonic day (E) 18.5, postnatal day (P) 0 and P14] (Modamio-Høybjør et al., 2007). Recently, a previously unreported mouse transcript variant 3 (NM_001289436.1) was predicted that includes a new exon (exon 3b) and changes the reading frame compared with that of the original mouse transcript. This variant is predicted to use an alternative downstream start codon (at the 5′ end of exon 7 of the long transcript), which would produce a shorter isoform (NP_001276365.1) corresponding to the last 155 aa of the long isoform. This exon 3b has been previously reported in mouse (Modamio-Høybjør et al., 2007); however, western-blot analysis did not detect this 155 aa isoform in the mouse inner ear (Modamio-Høybjør et al., 2007).

CCDC50 is an effector of epidermal growth factor (EGF)-mediated cell signalling as it is phosphorylated and ubiquitinated upon EGF stimulation (Blagoev et al., 2004; Kratchmarova et al., 2005). Moreover, CCDC50 is an inhibitor of the epidermal growth factor receptor (EGFR), mediating its endocytosis and playing a crucial role in the regulation of the amount of EGFR on the cell surface membrane (Tashiro et al., 2010, 2006). Therefore, it has been hypothesised that if it plays a similar role as other EGFR effectors, tyrosine phosphorylation of CCDC50 may regulate endosomal trafficking of EGFR (Zhang et al., 2005), which is thought to have a role in cochlear development and differentiation in rats (Zine et al., 2000). In addition, CCDC50 has been reported to be a negative regulator of the NF-κB and Fas signalling pathways as it interacts with the cytoplasmic zinc finger protein A20, and both proteins inhibit NF-κB activation (Kameda et al., 2009; Tsukiyama et al., 2012). Furthermore, it has recently been described that CCDC50 is an autophagic cargo receptor that negatively regulates interferon responses (Hou et al., 2021a,b).

CCDC50 is ubiquitously expressed in mice (Skvorak et al., 1999). Levels of CCDC50 within the cochlea vary dynamically from embryonic stages onwards when expressed in differentiating cells within various parts of the cochlea – such as in the otic mesenchyme, in cells lining the lumen of the cochlear duct and in nerve fibres of the spiral ganglion – where the protein colocalises with microtubule-based structures. In the mature cochlea, CCDC50 is only expressed in the stria vascularis, the pillar cells and the vestibular sensory epithelia. Furthermore, it has been shown that CCDC50 colocalises with microtubules of the mitotic apparatus in NIH 3T3 cells (Modamio-Høybjør et al., 2007).

Mutations in *CCDC50* cause moderate-to-profound SNHL (Modamio-Høybjør et al., 2007; Vona et al., 2014; Iwasa et al., 2016; Sommen et al., 2016). Our team reported the first DFNA44 case of a Spanish family segregating bilateral, symmetrical and progressive sensorineural hearing loss. At onset, the hearing loss was moderate in the low-to-mid frequencies during the first decade of life and later it progressed to profound deafness affecting all frequencies. This family has a tandem duplication of 8 bp (NM_174908.4: c.866_873dup) that segregates with the hearing impairment. This mutation leads to a frameshift that replaces the last 15 aa of the protein with a novel 36 aa sequence [NP_777568: p.(Phe292Hisfs*37)]. Other missense and nonsense mutations in *CCDC50* have also been reported to cause moderate-to-profound SNHL (Vona et al., 2014; Iwasa et al., 2016; Sommen et al., 2016).

Here, we report a previously unreported frameshift mutation in *CCDC50* [c.828_858del, p.(Asp276Glufs*40)] causing non-syndromic hearing impairment in a second Spanish family. To study the pathological mechanisms of the hearing loss owing to *CCDC50* mutations, we analysed two loss-of-function *Ccdc50* mutant mouse lines, which showed no hearing impairment up to 6 months of age in both heterozygous and homozygous mutant mice. However, the assessment of the two frameshift mutations identified in the Spanish families and of a set of artificial mutants in transfected cells revealed that only those carrying a specific sequence in their protein tail led to an abnormal protein distribution within the cell cytoplasm. Therefore, we suggest that the mechanism of pathogenesis underlying DFNA44 may be caused by a dominant-negative or gain-of-function effect of the CCDC50 mutant proteins.

## RESULTS

### Identification of a novel mutation in *CCDC50* causing SNHL

**Fig. 1.**
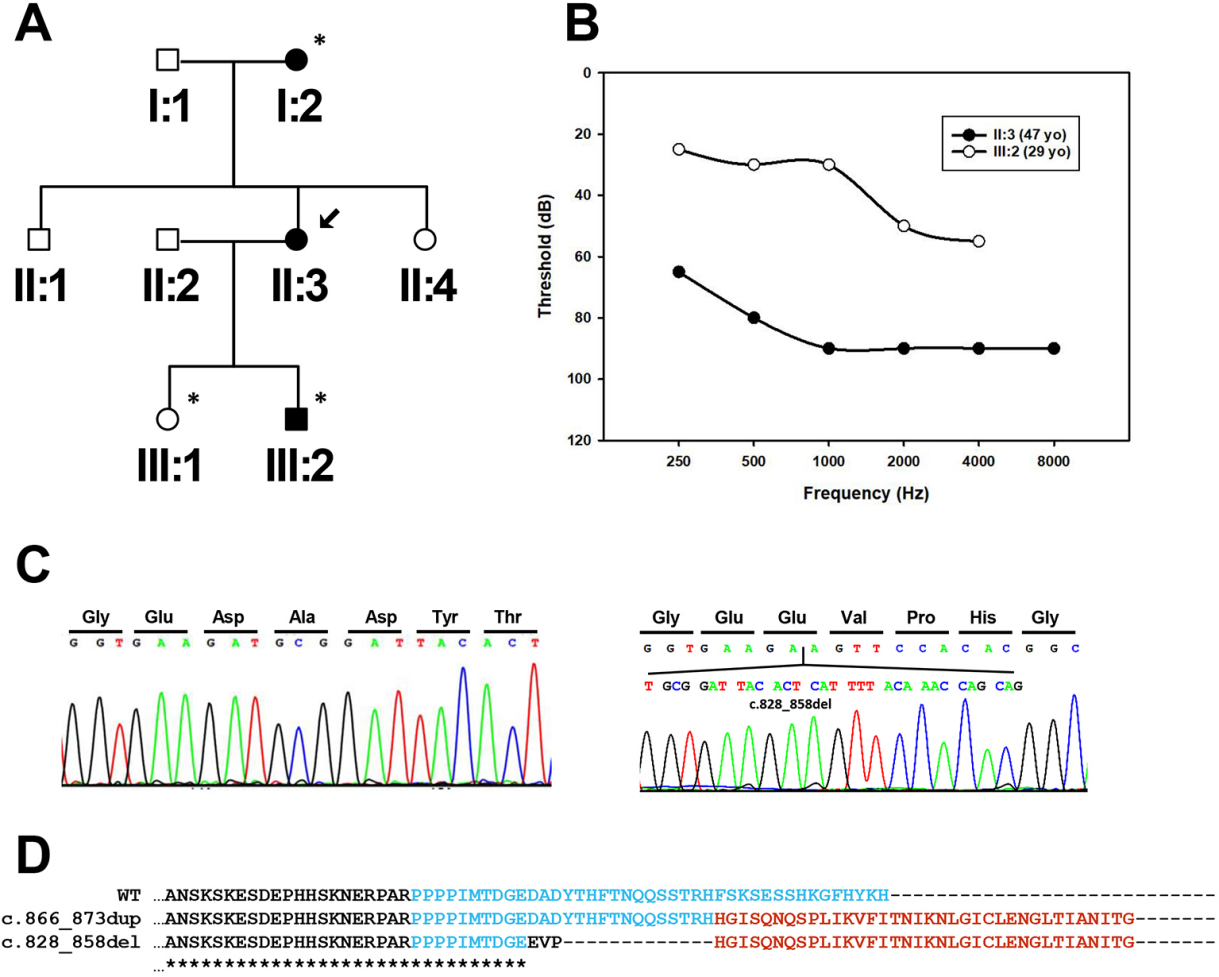
**Pedigree and audiogram of the family with the c.828_858del mutation.** (A) Pedigree of the Spanish family with the c.828_858del mutation in *CCDC50*. The patient analysed by OTO-NGS-panel V1 is indicated with an arrow. The individuals sequenced by Sanger sequencing to confirm segregation of the mutation are marked with asterisks. (B) Pure tone audiometry of subjects II:3 and III:2 showing air conduction thresholds at 250, 500, 1000, 2000, 4000 and 8000 Hz. The values represented are the average of the hearing threshold for the right and left ears. yo, years old. (C) Electropherograms showing the wildtype *CCDC50* exon 11 (left) and the c.828_858del mutation identified in patients II:3 and III:2. (D) Alignment of the C-terminal region of the wildtype CCDC50 protein and the c.866_873dup (p.Phe292Hisfs*37) and c.828_858del (p.Asp276Glufs*40) mutants. The sequence in cyan corresponds to the protein sequence encoded by exon 11 and the sequence fragments in red correspond to the aberrant protein tail of both mutants.

The family reported in this study has post-lingual progressive hearing loss that is consistent with an autosomal-dominant inheritance pattern (Fig. 1A). Audiological assessment of the affected member III:2 (29 years old) showed a moderate hearing loss, mostly affecting the high frequencies. His mother (individual II:3) presented a more severe phenotype, with severe hearing loss at frequencies higher than 1000 Hz (Fig. 1B).

Sequencing of 71 known deafness genes using our custom-designed next-generation sequencing (NGS) panel (OTO-NGS-panel V1) on DNA from individual II:3 identified a 31 bp deletion in heterozygosis in *CCDC50* (c.828_858del). The mutation is localised in exon 10 of the shorter transcript (NM_174908.4) and segregated with the hearing loss in the family (Fig. 1C). This variant also affects the longer transcript (NM_178335.3, containing exon 6), being localised in this case at exon 11. The deletion leads to a frameshift that skips the stop codon and generates an aberrant protein tail of 39 aa that replaces the last 31 aa of the wildtype protein (Fig. 1D). The c.828_858del mutation was classified as a pathogenic variant according to the American College of Medical Genetics and Genomics (ACMG) criteria (Kopanos et al., 2019; Richards et al., 2015). The variant was absent in the Genome Aggregation Database (GnomAD) (Karczewski et al., 2020), the Collaborative Spanish Variant Server (CSVS) database (Peña-Chilet et al., 2021) and the Deafness Variation Database (DVD) (Azaiez et al., 2018) (Table 1). The novel *CCDC50* variant c.828_858del has been deposited in ClinVar (accession number SCV002041905).

**Table 1.**
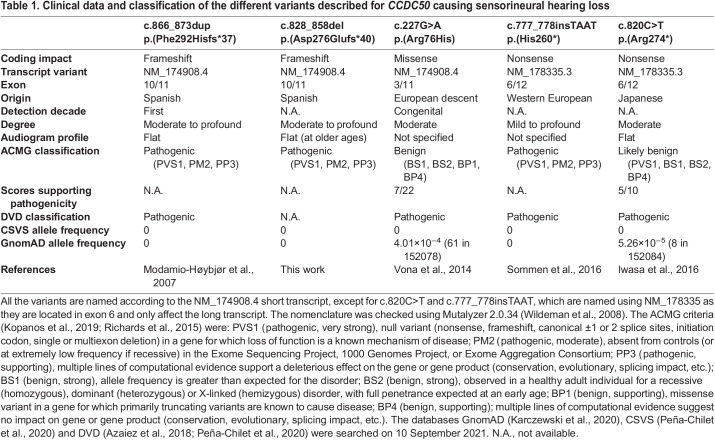
Clinical data and classification of the different variants described for *CCDC50* causing sensorineural hearing loss

### *Ccdc50* mutant mice have normal auditory brainstem response thresholds

**Fig. 2.**
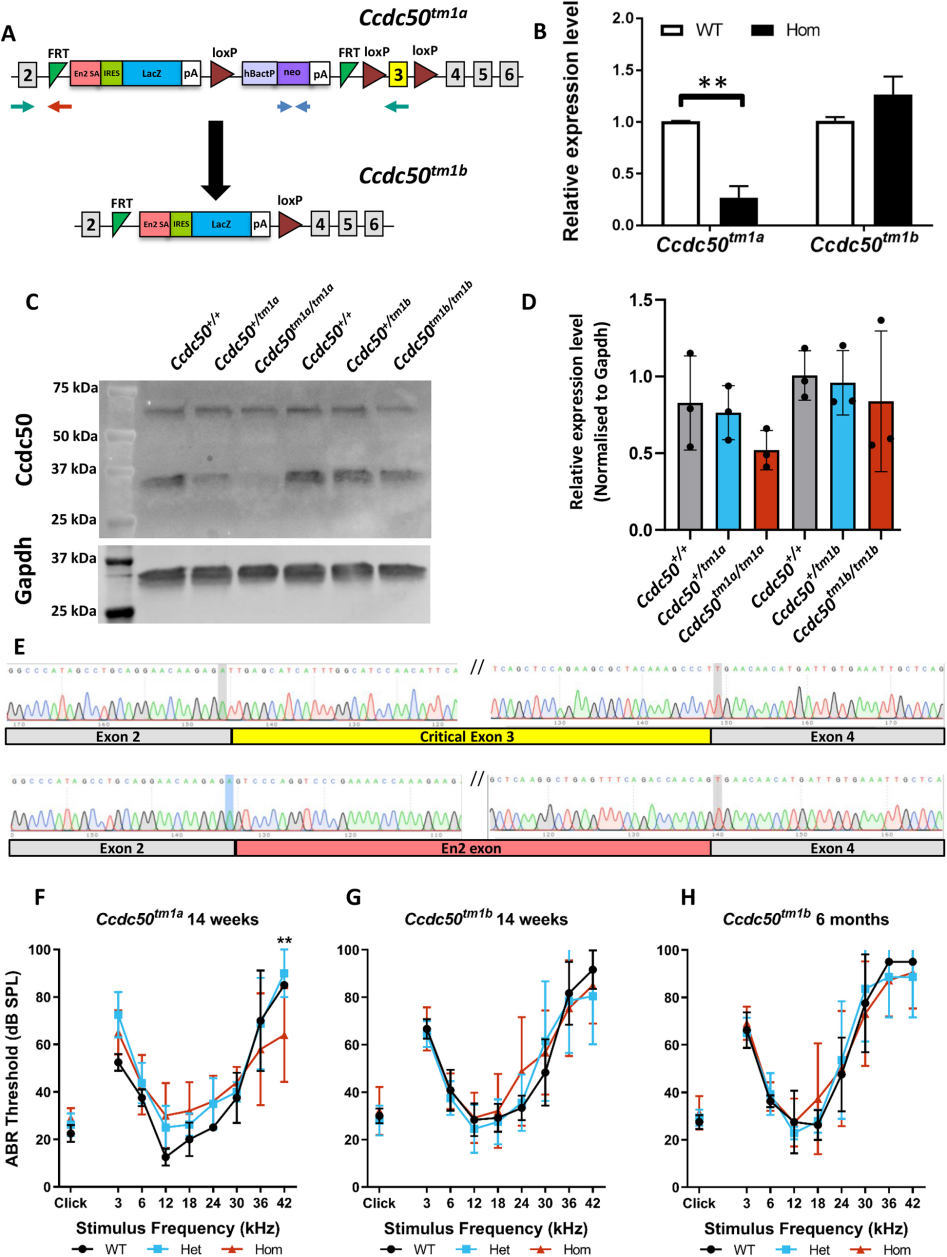
***Ccdc50* mutant mice show normal auditory thresholds.** (A) Design of the *Ccdc50^tm1a^* and *Ccdc50^tm1b^* alleles. A promoter-driven DNA cassette, containing *lacZ* (blue) and neomycin-resistance genes (neo, purple) with exon 3 (yellow) surrounded by loxP sites (red triangles), was inserted into intron 2 (top). Upon exposure to Cre recombinase, recombination of the loxP 1 and loxP 3 sites can occur, generating the *Ccdc50^tm1b^* allele in which exon 3 and part of the inserted cassette are deleted (bottom). Red and blue arrows indicate the location and orientation of the primers used for genotyping and nested PCR of cDNA. (B) RT-qPCR shows knockdown of *Ccdc50* transcription in 6-week-old brain tissue of *Ccdc50^tm1a^* homozygous (Hom) mice by 70%. In contrast, transcription is slightly increased in 6-week-old brain tissue of *Ccdc50^tm1b^* homozygous mice. *Hprt* was used as internal control and levels are normalised to wildtype (WT) levels (shown as 1.0 on the *y*-axis). Four WT and six homozygous *Ccdc50^tm1a^* animals were analysed, and four WT and four homozygous *Ccdc50^tm1b^* mice were used. Data are plotted as mean±s.d. Two-tailed unpaired *t*-test was performed: *Ccdc50^tm1a^*, **P=0.003; *Ccdc50^tm1b^*, *P*=0.11. (C) Western blotting performed on mouse brain samples. Two bands were revealed using the anti-CCDC50 antibody: one band of ∼35 kDa, corresponding to CCDC50, and another band of ∼70 kDa, which was still present upon treatment with increased concentrations of the reducing agents DTT and β-mercaptoethanol. Therefore, the larger band may correspond to non-specific binding of the polyclonal antibody used. Gapdh was used as a housekeeping protein. (D) Quantification of the CCDC50 protein band intensity, normalised to Gapdh. Relative expression levels are plotted for *Ccdc50^tm1a^* and *Ccdc50^tm1b^* wildtype mice (grey), heterozygotes (blue) and homozygotes (red). Data are plotted as mean±s.d. (*n*=3 for each genotype). The values for each mouse are plotted as black circles. (E) Sanger sequencing of cDNA. The results from only one WT (top) and one *Ccdc50^tm1b^* homozygote (bottom) are displayed here, but four mice for each genotype were sequenced with the same result: in all *Ccdc50^tm1b^* homozygous mice, exon 3 was deleted and, instead, part of the En2 sequence was present between exons 2 and 4 (*n*=4 for each genotype). (F-H) Auditory brainstem response (ABR) measurements. Mean ABR thresholds (±s.d.) for clicks and tone pips are plotted for WT (black), heterozygous (blue) and homozygous (red) mutant mice. Number of mice used: *Ccdc50^tm1a^*, WT=2, heterozygous (Het)= 4, Hom=5; *Ccdc50^tm1b^* at 14 weeks, WT=6, Het=10, Hom=12; *Ccdc50^tm1b^* at 6 months, WT=4, Het=7, Hom=11. All comparisons were tested for significance using a two-way ANOVA. Significant differences were only observed between *Ccdc50^tm1a^* heterozygotes and homozygotes at 42 kHz at 14 weeks of age (**P≤0.01). SPL, sound pressure level.

In order to investigate whether haploinsufficiency is the mechanism of hearing loss associated with *CCDC50* mutations, we studied the *Ccdc50^tm1a^* mutant mouse, which has a large DNA cassette inserted into intron 2 of the *Ccdc50* gene to interrupt its transcription (Fig. 2A, upper diagram) (Skarnes et al., 2011). In mature mice aged 14 weeks, we found no difference in auditory brainstem response (ABR) thresholds between homozygous mutants, heterozygotes and wildtype littermates (Fig. 2F). We assessed the amount of transcript from the *tm1a* allele, because these alleles can knock down rather than knock out transcription (White et al., 2013) owing to leaky suppression, which could explain the absence of hearing loss in the *Ccdc50^tm1a^* mutant mice. The *Ccdc50^tm1a^* homozygotes showed only partial knockdown of transcription, to 30% of wildtype levels, measured by real-time quantitative PCR (RT-qPCR) of brain tissue (Fig. 2B). Protein levels of CCDC50 were also decreased in the *Ccdc50^tm1a^* homozygotes, as revealed by western blotting (Fig. 2C,D).

**Fig. 3.**
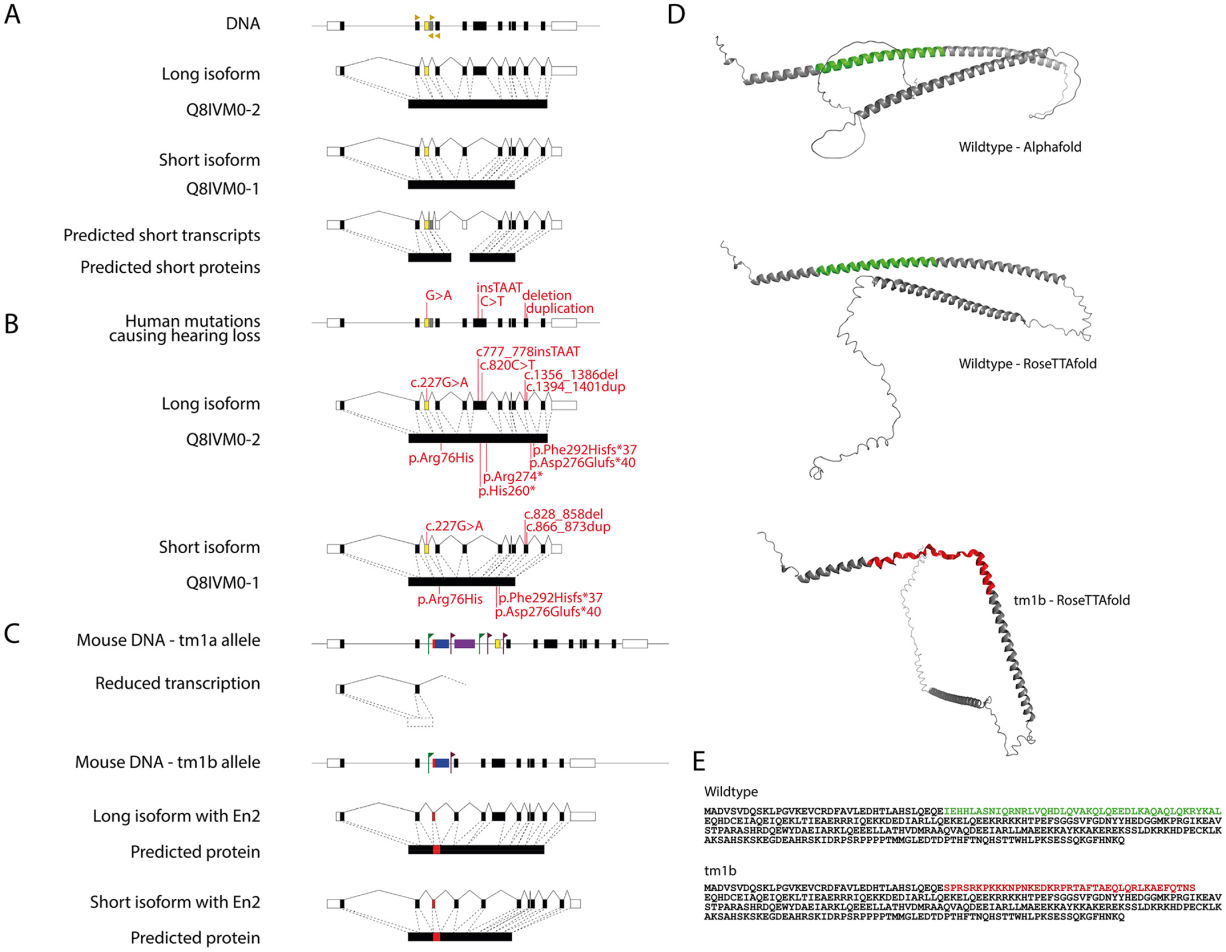
***CCDC50* isoforms, mutations and models.** (A) Exon structure of *CCDC50*, showing the two isoforms described in the human genome and the predicted short transcripts (NM_001289436.1; Modamio-Høybjør et al., 2007). Primer locations are marked by golden arrowheads. Exon 3 (the critical exon in the mouse *tm1a* and *tm1b* alleles) is coloured yellow, and exon 3b is coloured grey. Not to scale. (B) Human mutations causing hearing loss. Two of the reported mutations only affect the long isoform. (C) Mouse mutations described in this paper. The *tm1a* allele has a large DNA cassette inserted before exon 3 (red, blue and purple rectangles; loxP sites are marked with maroon flags and FRT sites with green flags). In the *tm1b* allele, the second part of the cassette has been excised by Cre-mediated recombination, leaving just the first section, which includes the *En2* splice acceptor site (red) and the *lacZ* gene (blue). Inclusion of the *En2* splice acceptor sequence up to a cryptic splice site results in restoration of the reading frame, and the two predicted transcripts are shown with red, for which the *En2* sequence is included in place of exon 3. (D) Models of CCDC50, showing the AlphaFold and the RoseTTAFold predictions of the wildtype protein. Exon 3 is located in the middle of the first α-helix, shown in green. The lower model shows the structure predicted from the mutant sequence, with the aberrant En2 section (red) in place of exon 3. (E) The two protein sequences used for modelling, based on UniProt Q810U5.

To generate a more severe allele, we produced the *Ccdc50^tm1b^* allele by breeding *Ccdc50^tm1a^* mice to *Hprt^Tg(CMV-Cre)Brd/Wtsi^* mice, which express Cre recombinase constitutively (Fig. 2A, lower diagram; Fig. 3C). Cre recombinase recombines DNA between loxP sites, leading to deletion of exon 3, which is present in all protein-coding splice variants of the *Ccdc50* gene. The relative expression levels of *Ccdc50* mRNA were measured in RNAs extracted from the brains of *Ccdc50^tm1b^* homozygous mice and littermate wildtype controls, and surprisingly the transcription in homozygotes was slightly upregulated. Therefore, in order to verify that exon 3 was deleted, the region between exons 2 and 4 in the *Ccdc50* transcript was sequenced in *Ccdc50^tm1b^* homozygous and littermate control mice, revealing the expected deletion of exon 3 but also retention of part of the initial *En2* sequence of the inserted cassette, ending in a novel splice site (Fig. 2E). Although the loss of exon 3 should introduce a frameshift, the 115 bp of *En2* sequence restores the frame of the transcript and could permit translation, resulting in a protein product with 39 residues of En2 in place of that coded by exon 3 of *Ccdc50*. We determined by western blotting that the CCDC50 protein was detected in both *Ccdc50^tm1b^* heterozygotes and homozygotes (Fig. 2C,D). However, this does not mean that the resulting aberrant CCDC50 protein is functional. The protein product of the wildtype exon 3 is 43 aa long and is part of an α-helix, whereas the inserted En2 sequence is shorter and from a disordered region. The wildtype mouse CCDC50 protein was modelled using RoseTTAFold (Baek et al., 2021) and compared with the model that included the inserted En2 sequence in place of the protein product of exon 3 (Fig. 3). The predicted change to the protein structure suggests that it would be unlikely to function normally.

Auditory function was investigated by ABR in *Ccdc50^tm1b^* mutants and littermate controls at 14 weeks and 6 months of age. No difference in ABR thresholds was observed between *Ccdc50^tm1b^* homozygotes, heterozygotes and control littermate mice at either age (Fig. 2G,H). These results suggest that the disruption of the *Ccdc50* gene does not lead to impaired ABR thresholds in mice, even in homozygotes in which an abnormal protein is expected to be produced. The original Spanish family described showed hearing impairment as early as 8 years of age (Modamio-Høybjør et al., 2003), so we would have expected to see signs of hearing loss by the age of 6 months in mice if there was a similar rate of progression compared with humans.

**Fig. 4.**
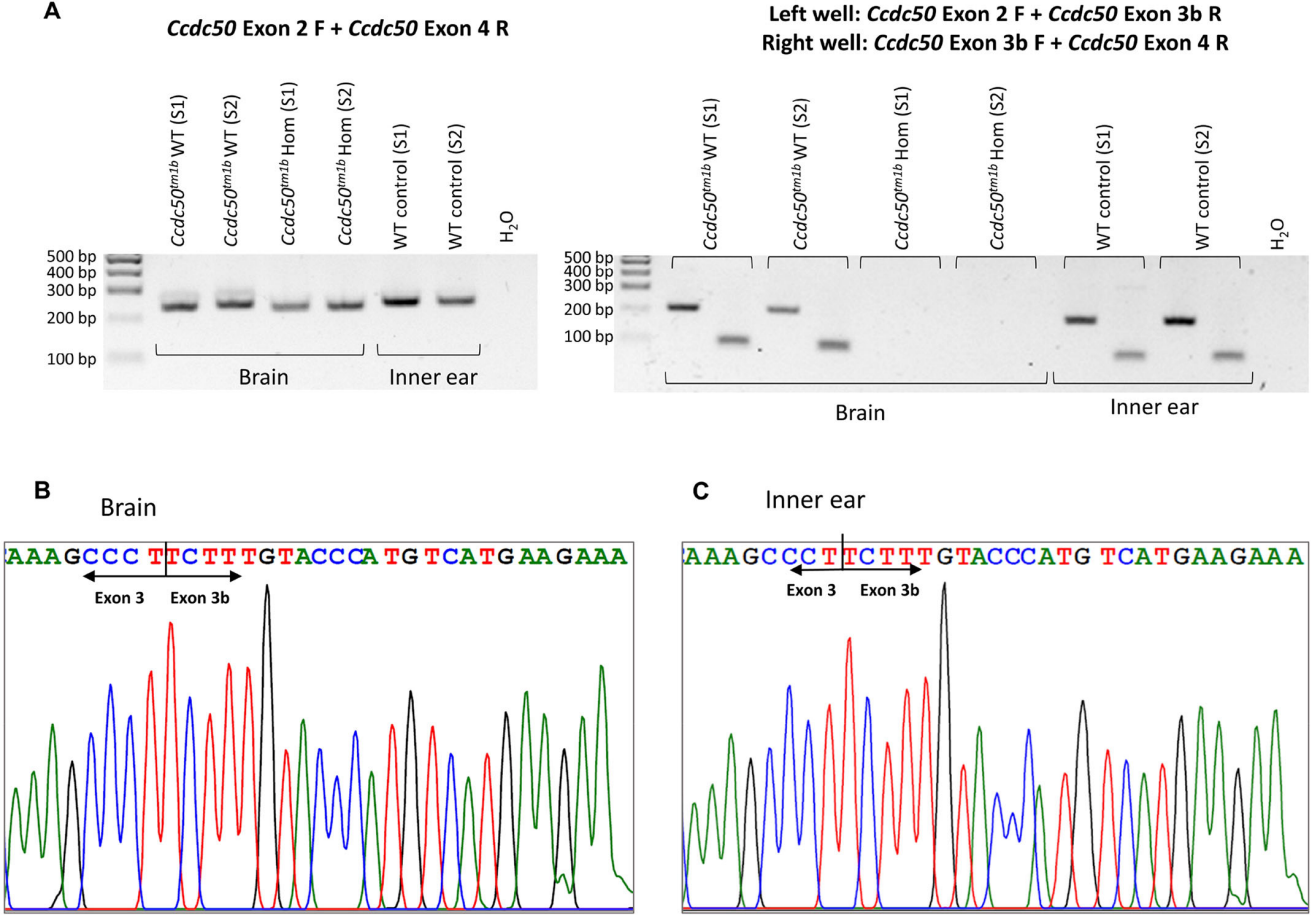
**Expression of *Ccdc50* transcript variant 3 in the mouse brain and inner ear.** (A) Nested PCR results in wildtype (WT) and homozygous (Hom) mice. Two samples were processed per genotype (S1 and S2). The primers used for each reaction are indicated above the gel. The first PCR product was smaller in the homozygotes than in the wildtypes as expected, as exon 3 (127 bp) and exon 3b (46 bp) were replaced by the 115 bp *En2* sequence (115 bp) in the mutant allele. For the second PCR, two different primer pairs were used, with one primer complementary to exon 3b in both reactions. The absence of amplification in the *Ccdc50^tm1b^* homozygotes indicates that this exon had been deleted. (B,C) Electropherograms indicating the expression of exon 3b in the brain (B) and the inner ear (C) of wildtype mice.

The design of the *Ccdc50^tm1a^* allele includes the insertion of a loxP site at position 16:27225814, downstream of exon 3, the critical exon. This position is at the 3′ end of exon 3b (16:27225771-27225816), and thus both exon 3 and exon 3b are likely to be absent in the *Ccdc50^tm1b^* allele. We therefore investigated the new predicted transcript by cDNA amplification using nested PCR. In wildtype mice, primers designed to amplify from exon 3b generated a PCR product of the expected sizes as previously described (Modamio-Høybjør et al., 2007), whereas no product was detected using cDNA from the *Ccdc50^tm1b^* allele as template (Fig. 4A). Sanger sequencing confirmed the presence of exon 3b in both wildtype brain and inner ear tissue samples (Fig. 4B,C). This experimental evidence supports the inclusion of exon 3b in the transcripts and confirms that it is deleted in the *Ccdc50^tm1b^* allele as predicted.

### The c.866_873dup and c.828_858del mutations generate identical protein tails and generate CCDC50 aggregates in NIH 3T3 cells

**Fig. 5.**
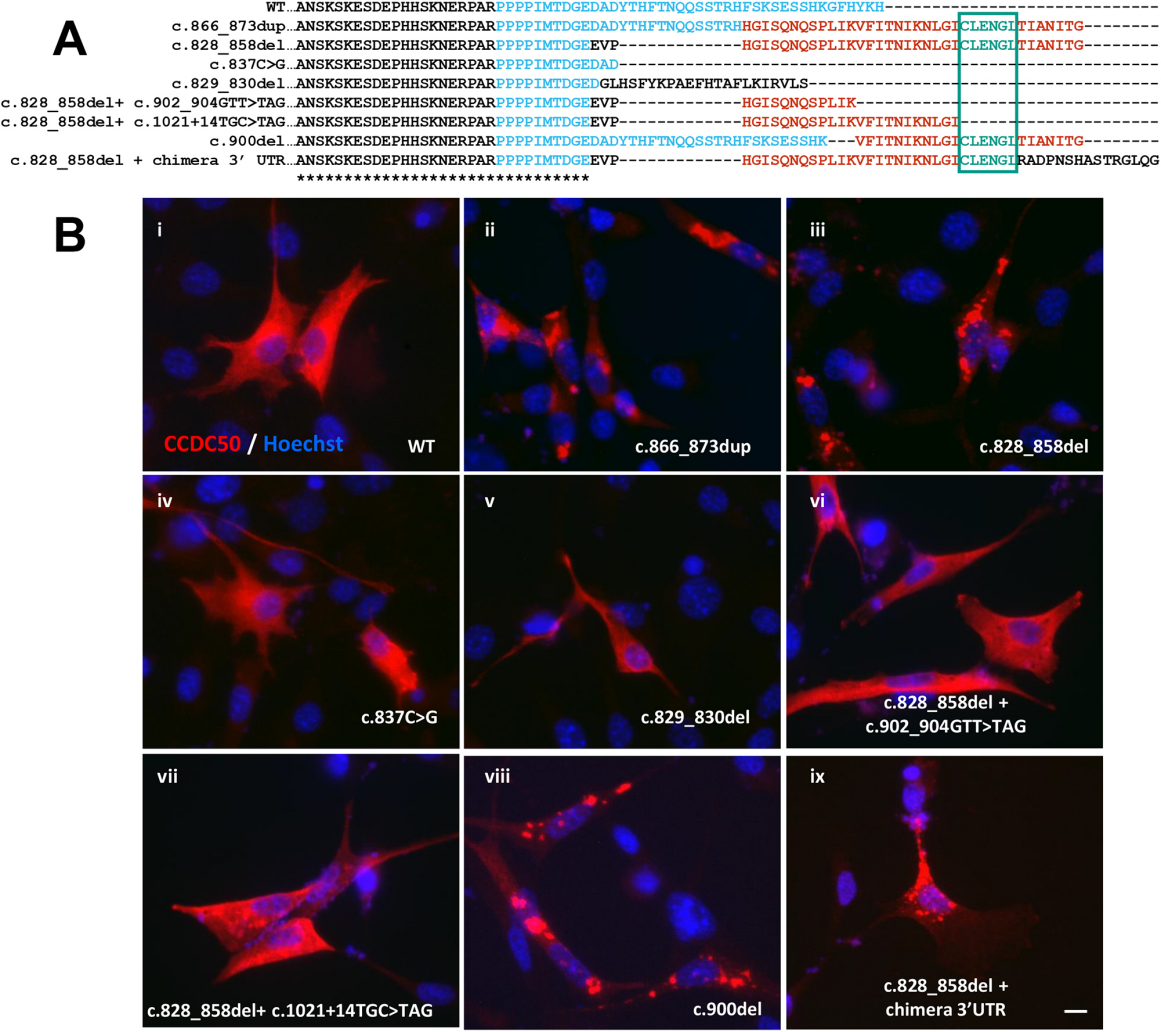
**Sequence alignment and subcellular localisation of CCDC50 mutants.** (A) Alignment of the C-terminal fragment of wildtype (WT) protein and the different mutants. The sequence in cyan corresponds to the protein sequence encoded by exon 11 and the sequence fragments in red are fragments shared by different mutants. The teal box indicates the CLENGL amino acid sequence, shared by all the mutants with an aberrant phenotype. (B) Effect of *CCDC50* mutations on CCDC50 cellular distribution in transiently transfected NIH 3T3 cells. The wildtype protein (i) and c.837C>G (iv), c.829_830del (v), c.828_858del+c.902_904GTT>TAG (vi) and c.828_858del+c.1021+14TGC>TAG (vii) mutants, lacking the CLENGL region, were widely distributed through the cytoplasm (red). The two mutations identified in patients, c.866_873dup (ii) and c.828_858del (iii), and the artificial c.900del (viii) and c.828_858del+chimera 3′ UTR (ix) mutations containing the CLENGL region showed an aggregated distribution of the protein, indicating that these six amino acids are essential for protein aggregation. Images are representative of three independent experiments. Scale bar: 25 μm.

In the absence of a recognisable phenotype in the *Ccdc50* mutant mice, we developed *in vitro* experiments to gain insights into the pathophysiology of the identified human mutations. Both frameshift mutations, c.828_858del (p.Asp276Glufs*40) identified in this work and c.866_873dup (p.Phe292Hisfs*37) identified in the first Spanish family (Modamio-Høybjør et al., 2007), generate aberrant CCDC50 protein tails. Interestingly, pairwise alignment of the predicted protein sequence generated by each mutant revealed that both mutations lead to a shared aberrant C-terminal tail (Fig. 1D). We previously reported that the wildtype CCDC50 protein is homogeneously distributed throughout the cytoplasm in NIH 3T3 cells, whereas the c.866_873dup mutant leads to an aggregated perinuclear distribution (Modamio-Høybjør et al., 2007). Therefore, to determine whether the cellular phenotype of the c.828_858del mutant was similar to that of the c.866_873dup mutant, we performed cell transfection of NIH 3T3 cells followed by immunocytochemistry. We observed that the c.828_858del mutant phenotype was similar to the one already published, with aggregate formation close to the nucleus (Fig. 5Bii-Biii), in contrast to the uniform cellular distribution in cells transfected with the wildtype sequence (Fig. 5Bi).

### The CLENGL amino acid sequence appears to be essential for CCDC50 aggregation

In order to determine the minimum amino acid sequence stretch that could drive the abnormal protein distribution pattern, we generated a set of six artificial CCDC50 mutant proteins sharing different tail parts (Fig. 5A). The presence of the mutations was confirmed by Sanger sequencing of the targeted plasmids prior to transfection into NIH 3T3 cells (Fig. S1).

The six different artificial mutants were as follows. (1) The c.837C>G mutation produces a premature stop codon upstream of the wildtype stop codon and both human mutations. We generated this mutant to determine whether the absence of the protein C-terminal tail prevented the formation of the perinuclear aggregates. The distribution pattern of the c.837C>G mutant distribution pattern was similar to that of the wildtype protein (Fig. 5Biv). As this mutant is shorter than the wildtype protein and does not have an aberrant tail, we concluded that the aberrant protein tail is necessary to form the CCDC50 aggregates. (2) The c.829_830del has a tail completely different from that in the two pathogenic alleles. This mutant was intended to ask whether the particular sequence of the mutant proteins was required to form the protein aggregates or if any aberrant sequence would lead to the same phenotype. In this case, CCDC50 distribution was also similar to that of the wildtype protein (Fig. 5Bv), supporting the idea that the amino acid sequence forming the aberrant tail shared by both human mutations is necessary to form the aggregates. (3) The c.828_858del+c.902_904GTT>TAG mutant contains a shorter aberrant tail than that generated by the two human mutations c.828_858del and c.866_873dup. This tail corresponds to the N-terminal region of the aberrant tail (HGISQNQSPLIK). No abnormal protein distribution was observed in this mutant (Fig. 5Bvi), indicating that the essential sequence for protein aggregation is located in the most C-terminal region of the aberrant tail. (4) The c.828_858del+c.1021+14TGC>TAG mutant shares the N-terminal sequence of the aberrant tail and it is longer than that of the previous mutant (HGISQNQSPLIKVFITNIKNLGI). This mutant did not show an abnormal distribution pattern (Fig. 5Bvii), indicating that the region responsible for aggregate formation is even more towards the C-terminal. (5) The c.900del mutant generates a tail shorter than the tails of the pathogenic alleles but shares the C-terminal region (VFITNIKNLGICLENGLTIANITG). CCDC50 distribution in cells transfected with this mutant was similar to the distribution of proteins with the human mutations, with abnormal protein aggregation (Fig. 5Bviii). Thus, the phenotype of this mutant suggests that the abnormal C-terminal part of the tail contains the essential sequence for the CCDC50 aggregation. Finally, (6) the c.828_858del+chimera 3′ UTR leads to a protein containing a modification of the deletion in the human protein (HGISQNQSPLIKVFITNIKNLGICLENGLRADPNSHASTRGLQG), with the same N-terminal sequence of the aberrant tail generated by the human mutations (HGISQNQSPLIKVFITNIKNLGICLENGL) but with a different C-terminal end (RADPNSHASTRGLQG). This mutant also showed abnormal distribution of the protein (Fig. 5Bix).

By comparing the altered phenotype obtained for the different protein tails in these cells, we observed that only the c.900del and c.828_858del+chimera 3′UTR mutants, which contain the amino acid sequence CLENGL in their protein tails (Fig. 5A), showed similar cytoplasmic aggregates to those seen for c.828_858del and c.866_873dup human mutations. In the other mutants, c.837C>G, c.829_830del, c.828_858del+ c.902_904GTT>TAG and c.828_858del+c.1021+14TGC>TAG, CCDC50 was homogeneously distributed throughout the cytoplasm, behaving as the wildtype protein (Fig. 5Bi). Taken together, these data indicate that the six-amino-acid sequence (CLENGL) drives the abnormal protein aggregation.

## DISCUSSION

Hearing loss caused by *CCDC50* mutations is a very rare disorder. To date, only five mutations in this gene have been reported to be associated with DFNA44 hearing impairment (Table 1), and the pathogenicity of some of these variants is still uncertain. Therefore, we need to understand further the pathogenic mechanism behind this kind of hearing impairment to improve our understanding of the disease and help with variant prioritisation.

In this work, we present a novel mutation in *CCDC50* causing autosomal dominant progressive hearing loss in a Spanish family. This variant [c.828_858del, p.(Asp276Glufs*40)] lies in the C-terminal domain and leads to a frameshift producing an aberrant protein tail in the CCDC50 protein, which is longer than that of the wildtype. The progressive hearing impairment in both Spanish families, together with the normal auditory thresholds in *Ccdc50* mutant mice, strongly suggest that c.828_858del and c.866_873dup mutations exert their effect through a dominant-negative mechanism on the wildtype protein or a gain of function. This could either be achieved by competing for partner proteins or recruiting other proteins to the protein aggregates, as the mutant gene product appears to adversely affect the wildtype gene product. However, further studies are needed to determine whether the protein aggregates overlap with any subcellular organelle or whether a combination of multiple proteins forms them. It is worth considering that both human mutations preserve the coiled-coil domain, which is one of the main oligomerisation motifs in proteins (Burkhard et al., 2001), and so, it is likely that the mutant CCDC50 preserves its interactors. Furthermore, both mutations preserve the motifs interacting with ubiquitin (Penengo et al., 2006), which are binding sites for ubiquitinated EGFR.

As for the critical amino acid region necessary for the formation of the aggregates (CLENGL), there is not a specific class of amino acid overrepresented within that sequence. Furthermore, it is not possible to predict a functional domain from such a short sequence. The CLENGL sequence was launched in the peptide search tool of the UniProt Knowledgebase (UniProtKB), a database for collecting functional information on proteins. However, the CLENGL peptide was not found to be overrepresented in any specific family of proteins.

The other variants in *CCDC50* reported in the literature (Table 1) do not contain the CLENGL sequence in the protein tail. Therefore, different mutations in *CCDC50* may exert their pathogenic effect through a different mechanism. For example, the variant p.(Arg76His) (Vona et al., 2014) affects the coiled-coil domain, so it might affect the interaction of CCDC50 with other proteins. The other two variants [p.(His260*) (Sommen et al., 2016) and p.(Arg274*) (Iwasa et al., 2016)] affect only the long isoform as they are located in exon 6. Both are truncating mutations that generate a premature stop codon and it is possible that a protein with only partial function is produced, leading to a dominantly inherited hearing loss. However, haploinsufficiency appears unlikely to be the underlying mechanism because the *Ccdc50^tm1a^* homozygous mouse mutant, with only 30% of the normal transcript levels, which is less than the expected 50% of normal transcript levels in people carrying one null *CCDC50* mutation, has normal ABR thresholds as we report here.

In contrast, the *Ccdc50^tm1b^* allele led to an abnormal transcript that retained a part of the *En2* sequence in place of exon 3 and was predicted by modelling to disrupt the normal α-helix and change the overall protein structure of the protein. This predicted disruption of the protein led to normal hearing in homozygotes. Another derivation of the *Ccdc50^tm1b^* mouse mutant has recently been screened by the International Mouse Phenotyping Consortium (IMPC), and they also reported normal ABR thresholds but minor metabolic and haematological anomalies and an increased grip strength, indicating that this mutation does have an impact on other phenotypes (https://www.mousephenotype.org/data/genes/MGI:1914751).

*CCDC50* is ubiquitously expressed. However, the hearing impairment in both Spanish families was not matched to any other characteristics suggestive of a syndromic hearing loss. Therefore, it is possible that the mutant forms of CCDC50 recruit new interactors that are only essential in the inner ear but not in other tissues, or that genetic redundancy exists in the other organs where *CCDC50* is expressed, which can somehow make up for the lack of functional protein. Likewise, the CCDC50 protein is expressed during inner ear maturation and in the mature cochlea, the latter correlating with the patients' postlingual appearance of the hearing impairment. A previous example of this has been reported in the case of *EYA4*, a ubiquitously expressed gene that is also involved in inner ear development and the mature cochlea, mutation of which leads to post lingual non-syndromic hearing loss (Wayne et al., 2001).

The data provided in this work provide further evidence towards the pathogenicity of the two mutations identified in the Spanish families (c.866_873dup and c.828_858del). Following the ACMG guidelines, both mutations were classified as pathogenic. However, the evidence provided in this work contributes towards the validation of the pathogenicity of both mutations, as we add two new pathogenicity criteria: PS3 (pathogenic strong 3) – well-established *in vitro* or *in vivo* functional studies supportive of a damaging effect on the gene or gene product – and PP1 (pathogenic supporting 1) – cosegregation with the disease in multiple affected family members in a gene definitively known to cause the disease. Furthermore, one of the ACMG criteria used to classify some of the variants listed in Table 1 is that null variants in a gene for which loss of function is a known disease mechanism are predicted to be pathogenic (PVS1). However, the absence of an auditory phenotype in the *Ccdc50* mouse mutants does not support loss of function as the pathogenic mechanism. This can help improve variant classification in the genetic diagnosis of hereditary hearing loss, highlighting the importance of this study to determine the pathogenicity of a given variant.

Understanding the pathogenic mechanism of the *CCDC50* mutations in the two Spanish families can help develop therapeutic approaches. A possible approach would be gene suppression by splice-switching antisense oligonucleotides or RNA interference (RNAi) with siRNAs or miRNAs (Shibata et al., 2016; Yoshimura et al., 2019). Alternatively, a stop codon could be generated upstream in close proximity to the mutation, as the artificial truncating mutations in the C-terminal region of *CCDC50* do not appear to lead to any phenotype in transfected cells.

To explore the alterations in the inner ear owing to the mutations identified in both families, it would be of interest to generate knock-in murine models for these variants. Developing a *CCDC50* knock-in mouse that specifically replicates the aberrant protein tail of the two Spanish mutations will help us to understand further the mechanisms underlying DFNA44 hearing loss. This could ultimately lead to therapeutic approaches to delay or prevent the hearing loss caused by mutations in *CCDC50*.

## MATERIALS AND METHODS

### Ethics statement

Mouse studies were carried out in accordance with UK Home Office regulations and the UK Animals (Scientific Procedures) Act of 1986 (ASPA) under UK Home Office licences, and the study was approved by the King's College London Ethical Review Committee. Mice were culled using methods approved under these licences to minimise any possibility of suffering.

### Genotyping of mice and generation of the *Ccdc50^tm1b^* allele

*Ccdc50^tm1a(EUCOMM)Hmgu^* mice (abbreviated to *Ccdc50^tm1a^*) were obtained from the European Mouse Mutant Archive (EMMA). They were generated from a targeted embryonic stem cell carrying a knockout-first, conditional-ready allele on a C57BL/6N genetic background (Skarnes et al., 2011; White et al., 2013; http://www.informatics.jax.org/allele/MGI:4841765), in which a promoter-driven cassette including *lacZ* and neomycin-resistance genes were inserted into intron 2 of the *Ccdc50* gene (Fig. 2A). DNA was extracted from the tissue of ear-clips and used as the template for short-range PCR using the forward primer 5′-TGCTGTTATGTCAAGGATGGA-3′ and the reverse primer 5′-AAGCTGGCCACTGTTCTTTC-3′ for the wildtype allele, and the same forward primer and the reverse primer 5′-GAACTTCGGAATAGGAACTTCG-3′ for the mutant allele. The presence of the promoter-driven cassette was confirmed by checking the presence of the neomycin-resistance gene using the forward primer 5′-CAAGATGGATTGCACGCAGGTTCTC-3′ and the reverse primer 5′-GACGAGATCCTCGCCGTCGGGCATGCGCGCC-3′ (Fig. 2A).

The *Ccdc50^tm1b^* allele was generated by crossing mice carrying the *tm1a* allele to *Hprt^Tg(CMV−Cre)Brd/Wtsi^* mice also on a C57BL/6N background, which expresses Cre recombinase constitutively, resulting in recombination between loxP sites in the *Ccdc50^tm1a^* allele and excision of the cassette (Fig. 2A). The *tm1b* allele was genotyped using the same primers used for the *tm1a* allele. The CMV-Cre allele was bred out of the colony before phenotypic characterisation. The primers used to detect the wildtype sequence at the *Hprt* locus were: forward, 5′-CTTTCCTCATGCCCCAAAATCTTAC-3′, and reverse, 5′-ATGTAATCCAGCAGGTCAGCAAGA-3′. For the mutant *Hprt^Tg(CMV-Cre)Brd/Wtsi^* allele, the forward primer was the same as that for the wildtype, and the reverse primer was 5′-GCTATCAGGACATAGCGTTGGCTAC-3′. In order to confirm the correct recombination and conversion into the *tm1b* allele, primers for loxP (forward, 5′-ATCCGGGGGTACCGCGTCGAG-3′, and reverse, 5′-ACTGATGGCGAGCTCAGACC-3′) and *tm1b* (forward, 5′-CGGTCGCTACCATTACCAGT-3′, reverse the same as that for loxP) were used.

The *Ccdc50* mouse mutants are available from public archives (https://www.infrafrontier.eu/search?keyword=Ccdc50 or https://www.mousephenotype.org/data/genes/MGI:1914751#order).

### RNA extraction and RT-qPCR

In order to minimise RNA degradation, all surfaces and tools were cleaned with RNase AWAY spray (Molecular BioProducts, 7002). Whole inner ears of 5-week-old wildtype mice were dissected and snap frozen. RNA extraction was performed using the SPLIT RNA extraction kit (Lexogen) according to the manufacturer's instructions. Whole brains of 6-week-old mice were dissected, cut in half along the midline, before snap freezing in liquid nitrogen, and all the samples were stored at −80°C. RNA isolation was performed by the phenol-chloroform method after homogenisation of the half brain, and TRIzol reagent (Sigma-Aldrich) was used according to the manufacturer's protocol. RNA concentration was measured using a Nanodrop spectrophotometer (ND-1000, Thermo Fisher Scientific). The concentration of all samples was adjusted to that of the specimen with the lowest concentration. The DNAse I kit (Sigma-Aldrich, AMP-D1) was used to degrade any DNA residue in the samples before the generation of cDNA using the Superscript II Reverse Transcriptase kit (Invitrogen, 11904-018) for the *Ccdc50^tm1a^* samples, and the Precision Reverse Transcription Premix 2 (Primer Design, RT-Premix2-48) for the *Ccdc50^tm1b^* samples. RT-qPCR was carried out using probes from Applied Biosystems (*Hprt*, 4351370, and *Ccdc50*, 4351372) and reagents from Bio-Rad (SsoAdvanced Master mix, 1725281). *Hprt* was used as the internal control, and the *Ccdc50* TaqMan probe chosen was downstream of exon 3, spanning exons 5 and 6. The 2^−ΔΔCt^ method was used to calculate relative expression levels between littermate mice. Three technical replicates were used for each sample. The data are presented as the fold change in gene expression normalised to that of the internal control (*Hprt* gene) and relative to the control mice (Livak and Schmittgen, 2001). The data were normally distributed and were analysed by a two-tailed unpaired *t*-test.

### Sequencing

Sequencing was performed in order to verify the deletion of the critical exon in the *Ccdc50^tm1b^* mutant mice. New sets of primers were designed using Primer 3 upstream and downstream of the critical exon: on exon 2 (F: 5′-TGTGTAGAGATTTTGCCGTCC-3′) and on exon 4 (R: 5′-CTCAATGGTTAGCTTCTCCTGG-3′). The same cDNA used for the RT-qPCR was used as template for the PCR amplification. PCR products were incubated at 37°C for 30 min, followed by 15 min at 80°C with ExoProStar (Illustra, US77720V). Sanger sequencing was carried out by Source BioScience (Nottingham, UK), and the results were visualised using SnapGene Viewer.

### Nested PCR methods

To determine whether the transcript variant 3 (NM_001289436.1) was expressed in our mutants, we designed a nested PCR assay based on two rounds of amplification. For the first PCR, we used cDNA extracted from the inner ear and the brain as a template and primers located in exons 2 and 4 (forward primer, 5′-TGTGTAGAGATTTTGCCGTCC-3′, and reverse primer: 5′-CTCAATGGTTAGCTTCTCCTGG-3′). Then, using this PCR product as a template, we ran two PCR reactions in which a primer located in exon 3b was used as follows: *Ccdc50* exon 2 F+*Ccdc50* exon 3b R (5′-TCTTCATGACATGGGTACAAAGA-3′), and *Ccdc50* exon 3b F (5′-TCTTTGTACCCATGTCATGAAGA-3′)+*Ccdc50* exon 4 R. The product of these PCRs was visualised in an agarose gel and sequenced by Sanger sequencing (Fig. 4).

### Western blot

Half brains were homogenised in protein lysis buffer (0.2% SDS, 0.5M EDTA pH 8 and proteinase inhibitor cocktail) and lysates were centrifuged at 13,000 ***g*** for 15 min at 4°C. Supernatants were collected and the protein concentration was determined using the Pierce BCA Protein Assay Kit (Thermo Fisher Scientific) according to the manufacturer's instructions. 30 µg of protein was incubated at 70°C for 10 min in NuPAGE LDS Sample Buffer (4×) with 1 mM dithiothreitol (DTT) and run in a 10% SDS-PAGE gel (Mini-PROTEAN TGX, Bio-Rad). Wet transfer was performed into PVDF membranes (Trans-Blot Turbo RTA Transfer Kit, Bio-Rad) using the Trans-Blot Turbo Transfer System (Bio-Rad). Membranes were blocked in 5% non-fat milk in PBS for 2 h at room temperature. Blots were incubated overnight with primary antibodies [anti-mouse Gapdh (1:5000, mouse monoclonal antibody, Abcam, ab8245) and anti-mouse CCDC50 (1:1000, rabbit polyclonal antibody, Thermo Fisher Scientific, PA5-51563)] in 2% milk in PBS containing 0.1% Tween 20 at 4°C under constant gentle shaking. Membranes were washed three times with PBS containing 0.1% Tween 20 and incubated with secondary antibodies [goat anti-rabbit IRDye 800CW (LI-COR, 926-32211) and donkey anti-mouse IRDye 680RD (LI-COR, 926-68072)] diluted 1:2000 in 2% milk in PBS containing 0.1% Tween 20. Imaging was performed in an iBright 1500 Imaging System (Invitrogen) and the band intensities were quantified using the Gel Analyzer tool in Fiji.

### Modelling the CCDC50 protein

The RoseTTAFold algorithm (Baek et al., 2021) was used to model CCDC50 via the Robetta protein structure prediction service (https://robetta.bakerlab.org) with default settings. Both the wildtype and the mutant sequence were uploaded (based on the transcript ENSMUST00000100026; see Table S2), and five protein models were assessed from each. The AlphaFold prediction of CCDC50 (accessed via Uniprot; Q810U5) was downloaded for comparison (Fig. 3). The first predicted structure for each RoseTTAFold sequence is shown in Fig. 3, as the first wildtype structure most closely resembled the AlphaFold structure. Pymol (The PyMOL Molecular Graphics System, version 1.7, Schrödinger) was used to generate the images from the models.

### ABR measurement

Mice were anaesthetised with an intraperitoneal injection of ketamine (1 mg/g)/xylazine (0.01 mg/g) and ABRs were recorded as previously described (Ingham et al., 2011). Subcutaneous needle electrodes were placed over the right bulla (ground), left bulla (reference) and vertex (active). Broad band click stimuli (10 μs duration positive transient, 256 sweeps presented 42.6/sec) and tone pips of 3-42 kHz (5 ms duration, 1 ms rise/fall time, 256 sweeps at a fixed phase) were delivered free-field at increasing levels of intensity from 0 to 95 dB sound pressure level in 5 dB steps. Both the click-evoked and tone-evoked potentials were amplified, filtered (300 Hz-3 kHz) and averaged to produce the ABR waveform. Response thresholds for each stimulus were estimated from the resulting ABR waveforms and defined as the lowest sound level at which any recognisable feature of the waveform was visible. The numbers of mice used were as follows: *Ccdc50^tm1a^* at 14 weeks of age, wildtype=2, heterozygote=4, homozygote=5; *Ccdc50^tm1b^* at 14 weeks of age, wildtype=6, heterozygote=10, homozygote=12; *Ccdc50^tm1b^* at 6 months of age, wildtype=4, heterozygote=7, homozygote=11.

### Patient selection

Patients and healthy relatives were selected in Hospital Ramón y Cajal (Madrid, Spain). Clinical evaluation excluded environmental factors as the cause of the hearing loss in the probands, and physical examination did not reveal any evidence of syndromic features. The hearing level was evaluated through pure tone audiometry. Air conduction thresholds were determined at frequencies ranging from 250 to 8000 Hz according to standard protocols. This study was designed in compliance with the tenets of the Helsinki Declaration, and the ethics committee that approved patient enrolment and the human research was the Institutional Review Boards of Hospital Ramón y Cajal (IRB number: 288-17). All participants provided written informed consent before they participated in the study.

### Sample collection

Whole blood samples were obtained from all participants by venepuncture in 5 mM EDTA tubes, and genomic DNA was extracted using Chemagen MSM I (Magnetic Separation Module I, PerkinElmer, MA, USA) according to the manufacturer's instructions. DNA was quantified by using a Qubit 3.0 Fluorometer (Thermo Fisher Scientific, MA, USA).

### Genetic screening of families with hearing loss

OTO-NGS-Panel V1 is a custom gene panel developed in the Genetics Service of Hospital Ramón y Cajal. This NGS tool uses the HaloPlex technology (Agilent) to capture all exons and 25 bp of the flanking introns of 71 genes involved in hereditary hearing loss. This tool was used to screen 111 Spanish independent familial cases with autosomal-dominant sensorineural hearing loss. The libraries were sequenced on the Illumina MiSeq (Illumina, San Diego, CA, USA) and raw data were analysed using the bioinformatic pipeline on DNAnexus software (https://www.dnanexus.com/). This pipeline aligns the sequences against the reference human genome GRCh37/hg19 with BWAMEM 0.7 aligner (https://github.com/lh3/bwa) and uses FreeBayes v.9.9.13 (https://github.com/ekg/freebayes) and GATK Variant Annotator  (https://github.com/broadinstitute/gatk) for variant annotation. The resulting variants were analysed with ANNOVAR (https://wannovar.wglab.org/) and verified by Sanger Sequencing. For variant prioritisation, an allele frequency filter lower than 1% in the following databases was applied: GnomAD (https://gnomad.broadinstitute.org/), the international genome sample resource (IGSR, https://www.internationalgenome.org/data), the Exome Aggregation Consortium (ExAC; https://gnomad.broadinstitute.org/), the CSVS database (http://csvs.babelomics.org/) and the DVD (http://deafnessvariationdatabase.org/).

### Cloning and mutagenesis

Human *CCDC50* cDNA was amplified using primers containing the restriction sites of NotI and SphI (forward primer, 5′-AAAAGCGGCCGCGTCTCCCGCTGCTTTGGT-3′; reverse primer, 5′-AAAAGCATGCCAAAATGGCAGTTTACAAAGGTC-3′). The PCR product was cloned into the pCR2.1 vector using the TA Cloning Kit (Invitrogen) using equimolar concentrations of plasmid and insert. Later, the plasmid was digested with NotI and SphI diluted in SuRE/Cut Buffer H (Roche) by overnight incubation at 37°C. Finally, the 1384 bp DNA fragment containing the *CCDC50* sequence was cloned into the expression vector pIRES-hrGFP-1a (Stratagene). The mutations c.866_873dup, c.828_858del, c.837C>G, c.829_830del, c.828_858del+c.902_904GTT>TAG, c.828_858del+c.1021+14TGC>TAG, c.900del and c.828_858del+chimera 3′ UTR (Fig. 5) were introduced by site-directed mutagenesis using the Quick Change II Site-Directed Mutagenesis Kit (Agilent Technologies) according to the manufacturer's instructions using custom-designed mutagenic primers (Table S1). Mutagenesis products were digested with the DpnI enzyme for 1 h at 37°C and transformed into XL10-Gold Ultracompetent Cells. Finally, plasmids were extracted using the QIAprep Spin Miniprep Kit (QIAGEN) and plasmids were sequenced using the BigDye Terminator v3.1 Cycle Sequencing Ready Reaction Kit (Thermo Fisher Scientific, 4337455) in an ABI PRISM 3100 genetic analyser (Applied Biosystems) to confirm the presence of the different mutations (Fig. S1).

### Cell transfection

NIH 3T3 mouse embryonic fibroblast cells, also used in our previous study (Modamio-Høybjør et al., 2007), were cultured in Dulbecco's modified Eagle medium (Sigma-Aldrich) supplemented with 10% fetal bovine serum (FBS, Gibco), 1% glutamine and 1% penicillin/streptomycin. For cell lipofection, cells were seeded in flat-bottomed 24-well multi-tier plates (Falcon; BD Biosciences, San Jose, CA, USA) covered with 12 mm glass coverslips (Menzel-Glaser, Braunschweig, Germany), and incubated at 37°C in a humidified CO_2_ incubator until they reached 70-90% confluency for transfection. Cells were transiently transfected with 500 ng of each pIRES-hr-GFP-1a mutant construct using Lipofectamine 2000 (111668, Invitrogen) according to the manufacturer's protocol.

### Immunocytochemistry and imaging

Cells were fixed 48 h post transfection with 4% paraformaldehyde in phosphate buffer (0.1 M NaH_2_PO_4_ pH 7.04) for 10 min and briefly rinsed with PBS three times. Cells were permeabilised for 10 min on ice with 0.5% Triton X-100 in PBS. Cultures were blocked with 3% bovine serum albumin in PBS for 30 min, washed twice with PBS and incubated for 1 h at room temperature with a 1:100 dilution of an anti-CCDC50 polyclonal antibody. This antibody was custom made by injecting a rabbit simultaneously with two peptides, one corresponding to the N-terminal part of the mouse CCDC50 protein (peptide I, RIQEKKDEDIARLL) and the other to the C-terminal region (peptide II, NQHSTTWHLPKSES) (CovalAb) (Modamio-Høybjør et al., 2007). Cells were washed three times with PBS and then incubated for 1 h with an Alexa Fluor 594 goat anti-rabbit IgG (H+L) (1:1000, Invitrogen, A-11037). Cell nuclei were counterstained with 1 µg/ml Hoechst 33342 (Sigma-Aldrich). Samples were visualised in an inverted fluorescence microscope (Olympus IX81), and images were taken with a cooled CCD-F View II (Soft Imaging System) camera.

## Supplementary Material

10.1242/dmm.049757_sup1 Supplementary information
