# Supplementary material for: Insights into the pathophysiology of DFNA44 hearing loss associated with CCDC50 frameshift variants
Source: Dis Model Mech. 2023 Aug 17;16(8):dmm049757. doi: 10.1242/dmm.049757 (PMC10445743; doi:10.1242/dmm.049757)
Supplement: Supplementary information [file dmm-16-049757-s1.pdf]

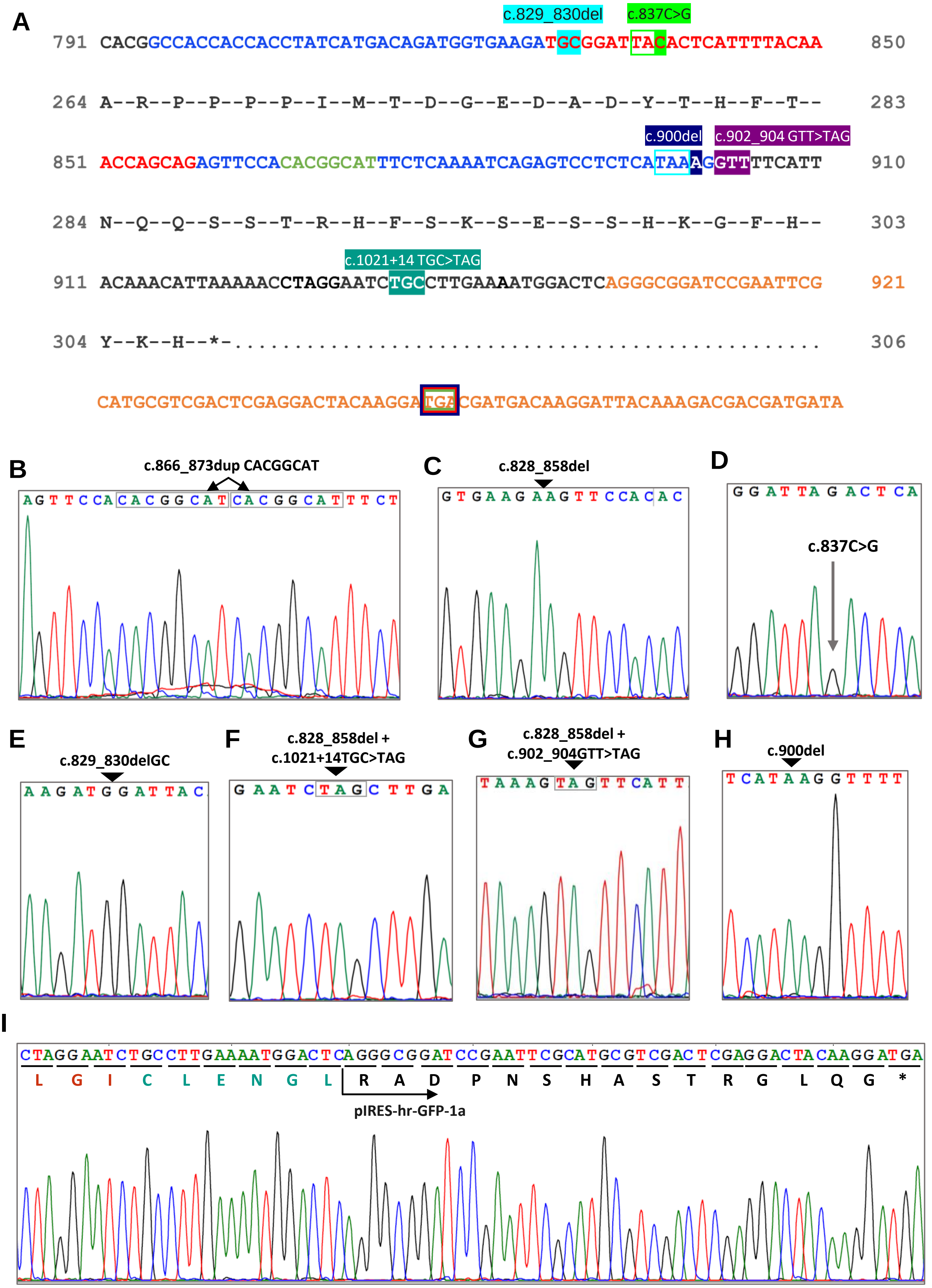

Disease Models & Mechanisms • Supplementary information

Fig. S1. Experimental design and Sanger sequencing validation of the artificial mutations. A) Sequence of the C-terminal region of the *CCDC50* NM_174908.4 transcript encoding the Ymer short isoforms. The first row corresponds to the cDNA, with position 1 being the A from the ATG initiation codon. The second row shows the protein sequence. Exons are indicated by alternating blue and black. The sequence indicated in orange corresponds to the plasmid in which the *CCDC50* cDNA was cloned (pIRES-hr-GFP-1a). The mutations identified in the Spanish families are indicated in red (c.828_858del) and green (c.866_873dup). All the mutations that we have artificially introduced are highlighted in the sequence. c.829_830del is highlighted in light blue, c.837C>G in green and c.900del in navy. Highlighted in purple is the GTT that changes to TAG in c.828_858del+c.902_904GTT>TAG mutant, and in teal is the TGC that changes to TAG in c.828_858del+c.1021+14TGC>TAG mutant. The stop codon used by each mutant is indicated by a square of the same colour as the mutation. B-I) Electropherograms of the Sanger sequencing verification of the different mutations. B,C) Human mutations. B) c.866_873dupCACGGCAT, C) c.828_858del, D-I) Artificial mutations introduced in the plasmid by site-directed mutagenesis. Mutations F,G,I were introduced using as a template the plasmid containing the 31 bp deletion. D) c.837C>G, E) c.829_830delGC, F) c.828_858del+c.1021+14TGC>TAG, G) c.828_858del +c.902_904GTT>TAG, H), c.900del, I) c.828_858del+chimera 3' UTR.

Table S1. Primers used for site-directed mutagenesis. F: forward, R: reverse.

Mutation	Primer (F/R)	Primer sequence (5'-3')
c.828_858del	F	AGTTCCACACGGCATTTC
	R	TCTTCACCATCTGTCATGATAG
c.866_873dup	F	CACGGCATCACGGCATTTCTCAAAATCAGAGTCC
	R	TGGAACTCTGCTGGTTTGTAAAATGAGTG
c.837C>G	F	GTTTGTAAAATGAGTCTAATCCGCATCTTCACCATCTGTC
	R	GACAGATGGTGAAGATGCGGATTAGACTCATTTTACAAAC
c.900del	F	CATTTCTCAAAATCAGAGTCCTCTCATAAGGTTTTCATTACAAACATTAAAAA
	R	TTTTTAATGTTTGTAATGAAAACCTTATGAGAGGACTCTGATTTTGAGAAATG
c.829_830del	F	CCTATCATGACAGATGGTGAAGATGGATTACACTCATTTTACA
	R	TGTAAAATGAGTGTAATCCATCTTCACCATCTGTCATGATAGG
c.828_858del + c.902_904GTT>TAG	F	AGATTCCTAGGTTTTTAATGTTTGTAATGAACTACTTTATGAGAGGACTCTGATTTT
	R	CATTTCTCAAAATCAGAGTCCTCTCATAAAGTAGTTCATTACAAACATTAAAAACCT
c.828_858del + c.1021+14TGC>TAG	F	TTGCTATAGTGAGTCCATTTTCAAGCTAGATTCCTAGGTTTTTAATGTTTGT
	R	ACAAACATTAAAAACCTAGGAATCTAGCTTGAAAATGGACTCACTATAGCAA
c.828_858del + chimera 3' UTR	F	TCACCCAGTAATATTTGCCGTAGTGAGTCCATTTTCAAGGCAGATTC
	R	GAATCTGCCTTGAAAATGGACTCACTACGGCAAATATTACTGGGTGA

Disease Models & Mechanisms • Supplementary information

Table S2. DNA sequences.

>Ccdc50 exon 3 (capitals) - exon 3b (lowercase)_brain

GAGCATCATTTGGCATCCAACATTCAGCGGAACCGTCTGGTACAACATGATCTGCAGGTTGCTAAGCA
GCTCCAAGAGGAAGACCTCAAAGCCCAAGCTCAGCTCCAGAAGCGCTACAAAGCCCTtctttgtaccc
atgtcatgaagaaa

>Ccdc50 exon 3 (capitals) - exon 3b (lowercase)_inner ear

GAGCATCATTTGGCATCCAACATTCAGCGGAACCGTCTGGTACAACATGATCTGCAGGTTGCTAAGCA
GCTCCAAGAGGAAGACCTCAAAGCCCAAGCTCAGCTCCAGAAGCGCTACAAAGCCCTtctttgtaccc
atgtcatgaagaaa

>Wildtype sequence (long isoform)

ATAGGCGAGGGGAGGCGGTCTGGCTCGCACGCCTCTGCGTGCGGTGCAGCCTTGCAGCCCCCGCCCCC
CGCCGCCGCCGCCGCCGCCTTTCTGGCACCTCTCTCCCTCCGTACTGGACTCCGGTGCATTTCCGGCC
CGGGAGAGTCCGGCGTCCACCCGGGCCAGCCCGCGGGGTGGGCGTGCGCCGTGATCTCCGCGCGCCCG
AGGCCCAGCGGCCGCCATGGCCGACGTGAGTGTAGATCAGTCCAAGTTGCCGGGCGTGAAGGAAGTGT
GTAGAGATTTTGCCGTCCTGGAGGACCACACCCTGGCCCATAGCCTGCAGGAACAAGAGATTGAGCAT
CATTTGGCATCCAACATTCAGCGGAACCGTCTGGTACAACATGATCTGCAGGTTGCTAAGCAGCTCCA
AGAGGAAGACCTCAAAGCCCAAGCTCAGCTCCAGAAGCGCTACAAAGCCCTTGAACAACATGATTGTG
AAATTGCTCAGGAAATCCAGGAGAAGCTAACCATTGAGGCTGAGAGACGACGCATTCAGGAAAAGAAG
GATGAGGACATAGCACGTCTTTTGCAAGAGAAGGAGCTACAGGAGGAGAAAAGGAGGAAGAAACACAC
TCCAGAGTTTTCTGGGGGCAGTGTTTTTGGAGATAACTACTATCATGAGGATGGAGACCAGTCAAGGT
CAAGGAGGGACAGGGAACTGGGTTCTGGACACTCAAAGTTTTGTAGACTCCAAAACGATGGGAAGACT
GTAAGGCAGAAGGAGAAACCAAGGCATCAACAGAAGAACTTGGAAGATCTGGAAGAACACCACCCATC
AGAGAGGTCCCTGCCTTCTGCTAGCTGGGGCAGAGGGAGGGATGGTGCCCAGACTGCCTGTGAGCAGC
AGGAAAGAAAGCGATCTGGTCAGGAGAGGCTCTGGAAATCTCCACTTCCGAAGATCCGCGGGGAGGTA
TTTCTGAGCATTGACTCTGAAGACTGGGAAGCTGACTGGAGCTCTGGAACCCGGAGTCCAGAAAAGCA
GTCTCATCACCATGGCAGACTTTCACCCAAGTCTTCACAGAAAACAGGACTTCCCTGTAAGGAAATTG
TATATGGGCGGGACCTTGGGCAAGGTGACCACAGAGAAAGGAGACATAGGCCCAGGACTTCTCCCTTC
TCAGAAGATAAAGAACTTCGCCACCACCATGTTGCAGGAATGAAACCAAGAGGAATAAAAGAAGCTGT
CTCTACTCCAGCACGAGCAAGCCACAGGGACCAGGAGTGGTATGATGCTGAAATTGCCAGGAAATTGC
AAGAAGAAGAACTTTTGGCTACTCATGTGGACATGAGAGCAGCTCAGGTTGCCCAGGATGAGGAAATT
GCTCGACTTCTAATGGCTGAAGAAAAAAAAGCTTACAAGAAAGCCAAAGAGCGAGAAAAGTCATCTTT
GGACAAAAGGAAACATGACCCTGAATGCAAGTTAAAAGCAAAGTCAGCCCACTCAAAGTCAAAAGAGG
GTGATGAAGCACACCGCTCCAAGATTGACAGGCCATCAAGACCACCACCACCTACCATGATGGGCCTT
GAGGACACAGATCCCACCCATTTTACAAACCAGCACAGTACAACATGGCATCTTCCAAAGTCAGAGTC
CTCACAGAAAGGCTTCCATAACAAGCAGTAAAATAATACAAACAAAAAGGGATCGGCCTCGAAAATGG
ACTTCGTATAGCACATATTACTTCTTATAGCACATATTACTGAAAGATACAACATGCATTCCACGTTG
GCTGTTGTCCT

>With En2

ATAGGCGAGGGGAGGCGGTCTGGCTCGCACGCCTCTGCGTGCGGTGCAGCCTTGCAGCCCCCGCCCCC
CGCCGCCGCCGCCGCCGCCTTTCTGGCACCTCTCTCCCTCCGTACTGGACTCCGGTGCATTTCCGGCC

CGGGAGAGTCCGGCGTCCACCCGGGCCAGCCCGCGGGGTGGGCGTGCGCCGTGATCTCCGCGCGCCCG
AGGCCCAGCGGCCGCCATGGCCGACGTGAGTGTAGATCAGTCCAAGTTGCCGGGCGTGAAGGAAGTGT
GTAGAGATTTTGCCGTCCTGGAGGACCACACCCTGGCCCATAGCCTGCAGGAACAAGAGAgtcccagg
tcccgaaaaccaaagaagaagaaccctaacaaagaggacaagcggcctcgcacagccttcactgctga
gcagctccagaggctcaaggctgagtttcagaccaacagTGAACAACATGATTGTGAAATTGCTCAGG
AAATCCAGGAGAAGCTAACCATTGAGGCTGAGAGACGACGCATTCAGGAAAAGAAGGATGAGGACATA
GCACGTCTTTTGCAAGAGAAGGAGCTACAGGAGGAGAAAAGGAGGAAGAAACACACTCCAGAGTTTTC
TGGGGGCAGTGTTTTTGGAGATAACTACTATCATGAGGATGGAGACCAGTCAAGGTCAAGGAGGGACA
GGGAACTGGGTTCTGGACACTCAAAGTTTTGTAGACTCCAAAACGATGGGAAGACTGTAAGGCAGAAG
GAGAAACCAAGGCATCAACAGAAGAACTTGGAAGATCTGGAAGAACACCACCCATCAGAGAGGTCCCT
GCCTTCTGCTAGCTGGGGCAGAGGGAGGGATGGTGCCCAGACTGCCTGTGAGCAGCAGGAAAGAAAGC
GATCTGGTCAGGAGAGGCTCTGGAAATCTCCACTTCCGAAGATCCGCGGGGAGGTATTTCTGAGCATT
GACTCTGAAGACTGGGAAGCTGACTGGAGCTCTGGAACCCGGAGTCCAGAAAAGCAGTCTCATCACCA
TGGCAGACTTTCACCCAAGTCTTCACAGAAAACAGGACTTCCCTGTAAGGAAATTGTATATGGGCGGG
ACCTTGGGCAAGGTGACCACAGAGAAAGGAGACATAGGCCCAGGACTTCTCCCTTCTCAGAAGATAAA
GAACTTCGCCACCACCATGTTGCAGGAATGAAACCAAGAGGAATAAAAGAAGCTGTCTCTACTCCAGC
ACGAGCAAGCCACAGGGACCAGGAGTGGTATGATGCTGAAATTGCCAGGAAATTGCAAGAAGAAGAAC
TTTTGGCTACTCATGTGGACATGAGAGCAGCTCAGGTTGCCCAGGATGAGGAAATTGCTCGACTTCTA
ATGGCTGAAGAAAAAAAAGCTTACAAGAAAGCCAAAGAGCGAGAAAAGTCATCTTTGGACAAAAGGAA
ACATGACCCTGAATGCAAGTTAAAAGCAAAGTCAGCCCACTCAAAGTCAAAAGAGGGTGATGAAGCAC
ACCGCTCCAAGATTGACAGGCCATCAAGACCACCACCACCTACCATGATGGGCCTTGAGGACACAGAT
CCCACCCATTTTACAAACCAGCACAGTACAACATGGCATCTTCCAAAGTCAGAGTCCTCACAGAAAGG
CTTCCATAACAAGCAGTAAAATAATACAAACAAAAAGGGATCGGCCTCGAAAATGGACTTCGTATAGC
ACATATTACTTCTTATAGCACATATTACTGAAAGATACAACATGCATTCCACGTTGGCTGTTGTCCT

>Wildtype protein

MADVSVDQSKLPGVKEVCRDFAVLEDHTLAHSLQEQEIEHHLASNIQRNRLVQHDLQVAKQLQEEDLK
AQAQLQKRYKALEQHDCEIAQEIQEKLTIEAERRRIQEKKDEDIARLLQEKELQEEKRRKKHTPEFSG
GSVFGDNYYHEDGGMKPRGIKEAVSTPARASHRDQEWYDAEIARKLQEEELLATHVDMRAAQVAQDEE
IARLLMAEEKKAYKKAKEREKSSLDKRKHDPECKLKAKSAHSKSKEGDEAHRSKIDRPSRPPPPTMMG
LEDTDPTHFTNQHSTTWHLPKSESSQKGFHNKQ

>Protein with En2

MADVSVDQSKLPGVKEVCRDFAVLEDHTLAHSLQEQEsprsrkpkkknpnkedkrprtaftaeqlqrl
kaefqtnsEQHDCEIAQEIQEKLTIEAERRRIQEKKDEDIARLLQEKELQEEKRRKKHTPEFSGGSVF
GDNYYHEDGGMKPRGIKEAVSTPARASHRDQEWYDAEIARKLQEEELLATHVDMRAAQVAQDEEIARL
LMAEEKKAYKKAKEREKSSLDKRKHDPECKLKAKSAHSKSKEGDEAHRSKIDRPSRPPPPTMMGLEDT
DPTHFTNQHSTTWHLPKSESSQKGFHNKQ

Disease Models & Mechanisms • Supplementary information
